# Distribution of malaria parasite-derived phosphatidylcholine in the infected erythrocyte

**DOI:** 10.1128/msphere.00131-23

**Published:** 2023-08-22

**Authors:** Tansy Vallintine, Christiaan van Ooij

**Affiliations:** 1 Department of Infection Biology, Faculty of Infectious Disease, London School of Hygiene & Tropical Medicine, London, United Kingdom; University at Buffalo, Buffalo, New York, USA

**Keywords:** malaria, *Plasmodium*, host-pathogen interactions, phospholipids

## Abstract

Malaria parasites modify their host erythrocyte in multiple ways, leading to changes in the deformability, adhesiveness, and permeability of the host erythrocyte. Most of these changes are mediated by proteins exported from the parasite to the host erythrocyte, where these proteins interact with the host cell cytoskeleton or form complexes in the plasma membrane of the infected erythrocyte. In addition, malaria parasites induce the formation of membranous compartments—the parasitophorous vacuole, the tubovesicular network (TVN), the Maurer’s clefts and small vesicles—within the infected erythrocyte, a cell that is normally devoid of internal membranes. After infection, changes also occur in the composition and asymmetry of the erythrocyte plasma membrane. Although many aspects of the mechanism of export of parasite proteins have become clear, the mechanism by which these membranous compartments are formed and expanded is almost entirely unknown. To determine whether parasite-derived phospholipids play a part in these processes, we applied a metabolic labeling technique that allows phosphatidylcholine to be labeled with a fluorophore. As the host erythrocyte cannot synthesize phospholipids, within infected erythrocytes, only parasite-derived phosphatidylcholine will be labeled with this technique. The results revealed that phosphatidylcholine produced by the parasite is distributed throughout the infected erythrocyte, including the TVN and the erythrocyte plasma membrane, but not Maurer’s clefts. Interestingly, labeled phospholipids were also detected in the erythrocyte plasma membrane very soon after invasion of the parasites, indicating that the parasite may add phospholipids to the host erythrocyte during invasion.

**IMPORTANCE:**

Here, we describe a previously unappreciated way in which the malaria parasite interacts with the host erythrocyte, namely, by the transfer of parasite phospholipids to the erythrocyte plasma membrane. This likely has important consequences for the survival of the parasite in the host cell and the host organism. We show that parasite-derived phospholipids are transferred from the parasite to the host erythrocyte plasma membrane and that other internal membranes that are produced after the parasite has invaded the cell are produced, at least in part, using parasite-derived phospholipids. The one exception to this is the Maurer’s cleft, a membranous organelle that is involved in the transport of parasite proteins to the surface of the erythrocyte. This reveals that the Maurer’s cleft is produced in a different manner than the other parasite-induced membranes. Overall, these findings provide a platform for the study of a new aspect of the host-parasite interaction.

## INTRODUCTION

The symptoms of malaria are the result of the infection of erythrocytes by the malaria parasite. Successful infection requires modifications of the host erythrocyte that result in various changes in the host erythrocyte—these allow the parasite to evade the host immune system and elimination in the spleen and increase uptake of nutrients. Many of the best-known modifications (such as those leading to changes in the deformability of the host erythrocyte, its adhesive properties, and its ability to take up nutrients) are the result of the export of proteins from the parasite to the host erythrocyte (1 – 3). Although many questions about the mechanism of protein export remain, several key steps are now well characterized (4, 5).

**Fig 1:**
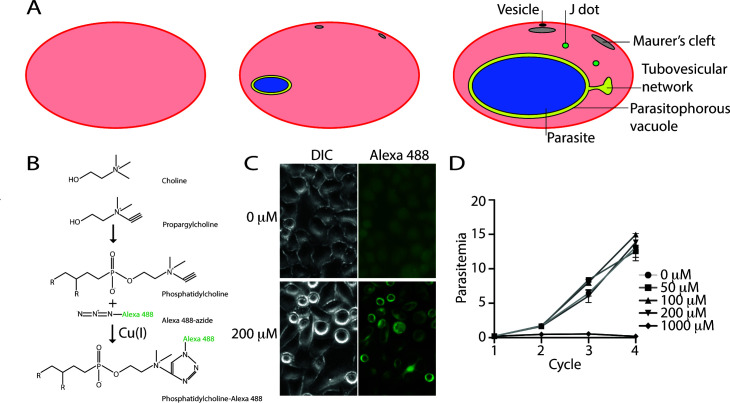
(A) Erythrocytes before and after infection with a *Plasmodium falciparum* parasite. The uninfected erythrocyte (left) is devoid of internal organelles, whereas an erythrocyte infected with a young parasite (center) contains multiple internal organelles. An erythrocyte infected with a late-stage parasite (right) contains further organelles. The origins of the lipids that form these membranes have not been identified. (**B**) Outline of the metabolic incorporation of propargylcholine into phosphatidylcholine and subsequent labeling with Alexa 488-azide using click chemistry. Propargylcholine can be metabolically incorporated into phosphatidylcholine, which can subsequently be conjugated to Alexa 488-azide using Cu(I) as catalyst, forming fluorescent phosphatidylcholine-Alexa 488. (**C**) Labeling of HeLa cells with Alexa 488-azide that had been cultured in the presence of the indicated concentration of propargylcholine for 48 h. (**D**) Growth of *P. falciparum* parasites in the presence of the indicated concentration of propargylcholine over four growth cycles. The experiment was performed in triplicate. Shown is one of two biological replicates.

In addition to changes in the protein content of the host erythrocyte, the parasite induces the formation of membrane-bound compartments in the erythrocyte—the exomembrane system (Fig. 1A)—that includes the Maurer’s clefts, small membranous compartments that are present underneath the host erythrocyte plasma membrane; the parasitophorous vacuole membrane (PVM), which surrounds the parasite and separates it from the host erythrocyte; the tubovesicular network (TVN), a little-studied extension of the parasitophorous vacuole membrane that protrudes into the host erythrocyte cytosol, frequently in a lollipop shape; and small vesicles that are often detected near the Maurer’s clefts (6). The origin of the membranes that form these structures is, in most cases, not known. Investigation of the invasion process has indicated that the nascent PVM that surrounds the parasite during and immediately after invasion is derived from host erythrocyte membrane (7), but the origin of the phospholipids required for the formation and expansion of the other internal membranes is unclear. The parasite can take up exogenous phospholipids (8 – 11), which the parasites may use to build these compartments, but the parasite also grows in the absence of exogenous phospholipids when the medium is supplemented with fatty acids (12 – 17). Hence, the parasite appears able to fulfill its phospholipid requirement solely with phospholipids that it synthesizes itself (18). In accordance with this, the parasite encodes all the enzymes required for the *de novo* synthesis of phospholipids (19). This includes the enzyme for the synthesis of phosphatidylcholine (choline/ethanolamine-phosphotransferase), which is essential and is located in the endoplasmic reticulum of the parasite, as determined in the rodent malaria parasite *Plasmodium berghei* (20)

In addition to the formation of membranous structures, the parasite induces changes in the composition and architecture of the erythrocyte plasma membrane. The distinct phospholipid asymmetry in the erythrocyte plasma membrane is altered in infected erythrocytes, with the level of phosphatidylethanolamine and phosphatidylcholineincreasing in the outer leaflet (10, 21 – 25). The phospholipid asymmetry in the plasma membrane of the erythrocyte is maintained by host erythrocyte flippase, floppase, and scramblase activity (26). However, after infection by a malaria parasite, the activity of flippase and scramblase changes, leading to the changes in phospholipid level in the different membrane leaflets (10). The fluidity of the membrane increases in the infected erythrocytes, which correlates with the age of the parasites (21, 27). In addition, the level of cholesterol and of sphingomyelin in the erythrocyte plasma membrane decreases as the parasite matures (28, 29). Furthermore, the acyl chain composition of the plasma membrane phospholipids is altered in infected erythrocytes compared to uninfected erythrocytes; palmitic acid (16:0) and oleic acid (18:1) become more prevalent in the plasma membrane of infected erythrocytes (22, 30 – 32). Interestingly, palmitic acid and oleic acid are the most prevalent fatty acids in phospholipids in the parasite, which has led to speculation that the parasite either modifies the phospholipids in the erythrocyte plasma membrane or transports phospholipids there (22, 30, 33).

Overall, it remains unclear how the internal membranes are formed and expanded in the host erythrocyte and how the parasite alters the composition of the host erythrocyte membrane. To gain insight into the origin of the phospholipids that form the compartments of the exomembrane system and the alterations of the host erythrocyte plasma membrane, we applied a metabolic labeling technique that allows newly synthesized phosphatidylcholine to be labeled using click chemistry. This revealed that parasite-derived phosphatidylcholine is distributed throughout the host erythrocyte but is not present in Maurer’s clefts.

## MATERIALS AND METHODS

### Parasites

All experiments were performed with the *Plasmodium falciparum* strain 3D7 and the *Plasmodium knowlesi* strain A1-H.1 (34). The parasites were maintained as described previously (35). Briefly, the parasites were maintained at 37°C in RPMI-1640 (Life Technologies) supplemented with 0.5% AlbuMax type II (Gibco), 50 µM hypoxanthine, and 2 mM l-glutamine. For *Plasmodium knowlesi,* the medium was further supplemented with 10% (vol/vol) horse serum. *P. falciparum* was incubated in the presence of 5% CO_2_, and *P. knowlesi* was incubated in an atmosphere of 90% N_2_, 1% O_2_, and 3% CO_2_. The hematocrit of the cultures was 2%–3%. Human erythrocytes were obtained from the National Blood Transfusion Service, UK, and from Cambridge Bioscience, UK.

*P. falciparum* parasites were synchronized by isolating late-stage infected erythrocytes on a discontinuous Percoll gradient. These isolated parasites were washed with RPMI-1640 and mixed with fresh erythrocytes to allow invasion. After 2–3 h, the remaining late-stage parasites were removed using a discontinuous Percoll gradient, and the resulting ring-stage culture was treated with 5% d-sorbitol to remove any remaining late-stage parasites (36, 37). To examine the parasites during and after invasion, schizonts were isolated on a Percoll gradient and incubated at 37°C in the presence of 25 nM ML10 to prevent egress. ML10 was then removed by centrifugation of the culture at 800 × *g* for 3 min and resuspension of the erythrocytes in warm RPMI-1640 with supplements. The parasites were then added to fresh erythrocytes, allowing for rapid and highly synchronous invasion (35, 38). To block invasion, cytochalasin D was added to the invading parasites at a concentration of 2 µM.

Growth of parasites was determined using a standard growth assay over four growth cycles. Synchronized ring-stage parasite cultures were adjusted to a parasitemia of 0.1% and maintained in a 24-well dish. Propargylcholine was added at the concentration indicated from a 100-mM aqueous stock solution. A 50-µL aliquot of the culture was removed every 48 h and mixed with 50 µL of 2× fixative solution (8% paraformaldehyde, 0.2% glutaraldehyde, and 2× SYBR Green in PBS). Fixed parasites were stored at 4°C until all samples were obtained. Starting from the third cycle, the medium was changed daily (the presence of propargylcholine was maintained throughout). When all samples had been obtained, the parasites were pelleted at ~2,000 × *g* for 1 min and the fixative was replaced with 1 mL PBS. A 200-µL aliquot was transferred to a 96-well plate, and the parasitemia was determined using a Thermo Attune N×T cytometer equipped with a Cytkick Max autosampler. Laser settings used for the detection of the erythrocytes and parasites were as follows: forward scatter 125 V, side scatter 350 V, blue laser (BL1) 530:30 280 V. The experiment was set up in triplicate, with two biological replicates.

Plasmid pS156 was introduced into 3D7 parasites using transfection of late-stage schizonts as described previously (39), followed by selection with 300 µg/mL G418. The plasmid pS156 was a kind gift of Sarah Tarr and Andrew Osborne (University College London). It was produced by replacing the gene encoding the Rex3-GFP fusion and *bsd* in the plasmid PFI1755c 1–61:GFP (40) with a gene encoding SBP1-mCherry and *neo*, respectively.

### Phospholipid labeling

Phosphatidylcholine was labeled using propargylcholine metabolic labeling and subsequent click chemistry as described previously (41, 42). Propargylcholine was added directly to the culture medium of parasites maintained in a 24-well dish; unless otherwise stated, the concentration used was 200 µM, diluted from a 100 mM aqueous stock solution. Parasites were harvested and washed with RPMI-1640 before resuspension in 1 mL fixative (4% paraformaldehyde, 0.1% glutaraldehyde in PBS) and incubation at room temperature for 1 h on a rotating wheel. It was noted that increasing the ratio of fixative to parasites improved the subsequent labeling. Parasites were pelleted (all spins in the procedure were at 2,000 × *g* for 1 min), and the fixative was replaced with 1 mL PBS. To label phosphatidylcholine containing propargylcholine with Alexa 488-azide, the parasites were pelleted, resuspended in 500 µL of freshly prepared labeling mix [40 mM Tris, pH 8.5, 2 mM CuSO_4_, 10 µM Alexa 488-azide, 2 µg/mL wheat germ agglutinin (WGA)-Alexa 647], 2 µg/mL Hoechst 33342 and 100 mM ascorbic acid (the ascorbic acid was always added last), and incubated at room temperature for 30 min on a rotating wheel. The parasites were then washed 6–8 times with 1 mL PBS. Fixed and labeled parasites were stored in PBS at 4°C. To prepare the parasites for microscopy, 1.5 µL of parasite pellet was mixed with 1.5 µL Vectashield mounting medium on a microscope slide. The mixture was covered with a coverslip and sealed with nail polish.

### Inhibitor treatment

To determine the effect of inhibitors on the transfer of parasite-derived phospholipids to the erythrocyte, parasites were synchronized as described above. Approximately 24 h after invasion, when the majority of the parasites contained visible hemozoin but only one nucleus still, propargylcholine was added to 200 µM and the parasites were treated with 1.25 µM Brefeldin A (from a 10-mM stock), 2 µM cytochalasin D (from a 2-mM stock), or 0.1% ethanol as solvent control. The erythrocytes were harvested the following day when the parasites in the sample treated with ethanol had reached the late schizont stage (and some rings were detected; no rings were detected in the other samples) and subsequently fixed, labeled with Alexa 488-azide, WGA, and Hoechst 33342 and imaged as described above.

### HeLa cell culture

HeLa cells were maintained in RPMI-1640 supplemented with 5% fetal bovine serum in an atmosphere of 5% CO_2_. Cells were maintained using periodic trypsinization and dilution. To label HeLa cells, the cells were seeded on round, acid-treated coverglasses in a 24-well dish and incubated in the presence of 200 µM propargylcholine for approximately 48 h, starting when the cells were ~40% confluent. The cells were then fixed and labeled with Alexa 488-azide similar to the fixation and labeling of erythrocytes described above, as described previously (41, 42). The coverglasses were placed cell-side down into 2–3 µL Vectashield and sealed with nail polish.

### Microscopy

Samples were imaged on a Nikon Ti-E inverted microscope with a Hamamatsu ORCA-Flash 4.0 Camera and Piezo stage driven by Nikon NIS-Elements version 5.3 software using the following excitation wavelengths for fluorescence image acquisition: 365 nm (Hoechst), 470 nm (Alexa 488), 580 nm (mCherry), and 635 nm (WGA). The images were deconvolved using Nikon NIS-Elements software (Richardson-Lucy, 20 iterations), and brightness and contrast were set using FIJI. The images were cropped using FIJI, and the file size was changed using Photoshop, and figures were produced using Illustrator. To measure the intensity of the staining, the line intensity tool in FIJI was used.

For confocal microscopy, samples were imaged on a Zeiss LSM 880 confocal microscope driven by Zen Black version 2.3 software. The emission wavelengths used were 462 nm (Hoechst), 548 nm (Alexa 488), and 637 nm (mCherry). Frame time was 11.3246 s. Brightness, contrast, and crop size were set in FIJI, the file size was changed using Photoshop, and figures were produced using Illustrator.

## RESULTS

### Malaria parasites replicate in the presence of propargylcholine

To determine whether the parasite distributes its own phospholipids within the host erythrocyte, we aimed to localize parasite-derived phosphatidylcholine by metabolically labeling phosphatidylcholine using propargylcholine. Propargylcholine is a derivative of choline that is modified with an alkyne group (Fig. 1B); this can be incorporated into phosphatidylcholine and subsequently allows a reporter molecule containing an azide group to be covalently linked to it using click chemistry (Fig. 1B). Labeling eukaryotic cells with propargylcholine is a well-established technique to investigate the intracellular distribution of phosphatidylcholine (41, 42) and has previously been applied to the investigation of the origin of lipids obtained by malaria parasites during the liver stage (43). We initially tested whether HeLa cells could incorporate propargylcholine and phosphatidylcholine could be visualized using Alexa 488-azide by culturing these cells in the presence or absence of propargylcholine and subsequently labeling these cells using the established protocol (41, 42). This revealed that HeLa cells that had been cultured in the presence of propargylcholine became brightly labeled with Alexa 488-azide, with a staining pattern very similar to that previously detected (42) (Fig. 1C).

To determine whether it would be possible to label malaria parasites with propargylcholine, we investigated the effect of propargylcholine on the growth of *Plasmodium falciparum* parasites. In the presence of propargylcholine at concentrations of 200 µM or below, the parasites replicated at a rate similar to that of untreated parasites (Fig. 1D). Subsequent experiments were, therefore, carried out using 200 µM propargylcholine unless otherwise stated.

### Parasite-derived phospholipids are transported to the erythrocyte plasma membrane

**Fig 2:**
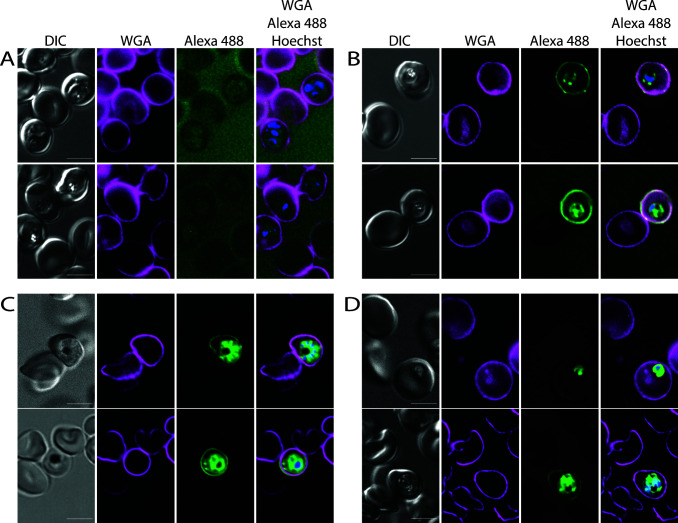
Metabolic labeling of erythrocytes infected with *Plasmodium* parasites with propargylcholine and stained with Alexa 488-azide. (**A**) Erythrocytes infected with *Plasmodium falciparum* were incubated without propargylcholine for 72 h and subsequently fixed and stained with Alexa 488-azide, wheat germ agglutinin, and Hoechst 33342. (**B and C**) Erythrocytes infected with *Plasmodium falciparum* were incubated in the presence of 200 µM propargylcholine for 72 h and subsequently fixed and stained with Alexa 488-azide, wheat germ agglutinin, and Hoechst 33342. Shown are trophozoites (**B**) and schizonts (**C**), clearly displaying labeling within the parasite and the erythrocyte membrane. No fluorescence was detected in uninfected erythrocytes. (**D**) Erythrocytes infected with *Plasmodium knowlesi* were incubated in the presence of 200 µM propargylcholine for 36 h and subsequently fixed and stained with Alexa 488-azide, wheat germ agglutinin, and Hoechst 33342. Note the distinct staining of the parasite and erythrocyte plasma membrane of infected erythrocytes and the absence of staining of uninfected erythrocytes. scale bar: 5 µm.

We next determined whether phospholipids can be visualized in parasites incubated in the presence of propargylcholine. As erythrocytes do not produce phospholipids (44), they will not incorporate propargylcholine into phospholipids, and hence, in erythrocytes infected with malaria parasites, any labeled phosphatidylcholine will have originated in the parasite. Phosphatidylcholine is the most abundant phospholipid in the parasite and makes up approximately 40%–50% of the phospholipids produced by the parasite (25, 30); hence, a large fraction of the parasite-derived phospholipids will contain propargylcholine. To label phosphatidylcholine, *P. falciparum* cultures were propagated either in the presence of propargylcholine or without added propargylcholine for 72 h (one and a half cycles), fixed, and then labeled with Alexa 488-azide. The plasma membrane of the erythrocytes was also stained with wheat germ agglutinin fused to Alexa Fluor 647 to provide a clear outline of the host erythrocyte. Infected erythrocytes in the culture maintained without propargylcholine did not display green fluorescence after fixation and staining, indicating that Alexa 488-azide labeling of erythrocytes or parasites requires propargylcholine (Fig. 2A). In contrast, parasites maintained in the presence of propargylcholine were fluorescently labeled (Fig. 2B and C). This labeling was detected within the parasites, as would be expected in growing parasites that are actively synthesizing phospholipids—late-stage parasites, especially segregated schizonts, were often very brightly labeled (Fig. 2C) as demand for newly synthesized phospholipids increases during this stage, owing to the formation of the plasma membrane for the invaginating parasite and the inner membrane complex (45). Furthermore, fluorescence was also detected in the erythrocyte plasma membrane, indicating that parasite phospholipids are transported to the host (Fig. 2B and C). In contrast, uninfected erythrocytes, which are not capable of phospholipid biosynthesis, were not fluorescent, indicating that active phospholipid metabolism is required for the incorporation of propargylcholine and subsequent labeling with the fluorophore. Decreasing the concentration of propargylcholine in the medium led to lower levels of fluorescence after fixation and labeling (Fig. S1). Similarly, when synchronized parasites were incubated in the presence of propargylcholine for just 3 h during the late stage of the erythrocytic cycle, only a very low level of fluorescence was detected in the infected erythrocytes as compared to parasites that had been labeled for nearly the entire erythrocytic cycle (Fig. S2), indicating that unincorporated propargylcholine was removed either in the initial washing step or subsequent steps of the procedure and the observed fluorescence does not represent propargylcholine that remained in the infected erythrocyte. No labeling of the host erythrocyte membrane was detected in the infected erythrocytes that had been labeled for only a short period.

To determine whether the export of phospholipids is common among *Plasmodium* spp., we repeated the labeling with erythrocytes infected with *Plasmodium knowlesi*. Similar to *P. falciparum*, fluorescence was detected in *P. knowlesi* parasites as well as the infected erythrocyte plasma membrane, but not in uninfected erythrocytes, indicating that the transport of parasite-derived phospholipids to the host erythrocyte plasma membrane is a property shared among *Plasmodium* spp. (Fig. 2D).

We aimed to obtain insight into the mechanism of the phospholipid transfer from the parasite to the host cell and, therefore, treated the parasites with Brefeldin A (BFA), an inhibitor of ER-to-Golgi transport, and cytochalasin D, an inhibitor of actin polymerization. To keep the inhibitor treatment of the parasites as short as possible, labeling of synchronized parasites with propargylcholine and the inhibitor treatment were initiated when the parasites were in the early trophozoite stage. The localization of parasite-derived phospholipids was subsequently determined when the parasites that had not been treated with inhibitor were in the late schizont stage. This revealed that in neither inhibitor-treated culture, parasite-derived phospholipid could be detected in the erythrocyte plasma membrane (Fig. S3). In the case of BFA-treated parasites, the staining was detected primarily in a compartment within the parasite that likely corresponds to the endoplasmic reticulum, whereas in the CD-treated parasites, the staining was detected throughout the parasites, with an accumulation in small compartment seen within parasites. These results implicate a role for anterograde exocytic transport and microtubule-based transport in the transport of the parasites-derived phosphatidylcholine from the parasite to the host cell. However, owing to long inhibitor treatment required and the strong effect the inhibitors have on the growth of the parasites, it is possible that the effect of the inhibitor may be indirect. Nonetheless, the finding that the infected erythrocytes in the inhibitor-treated cultures are not fluorescent indicates that there is no passive transfer of the phospholipid during the fixation or the staining procedure. Hence, the staining seen in the erythrocyte membrane in Fig. 2B and C represents active transport of the phospholipid in a process that potentially requires anterograde exocytic transport and actin filaments.

**Fig 3:**
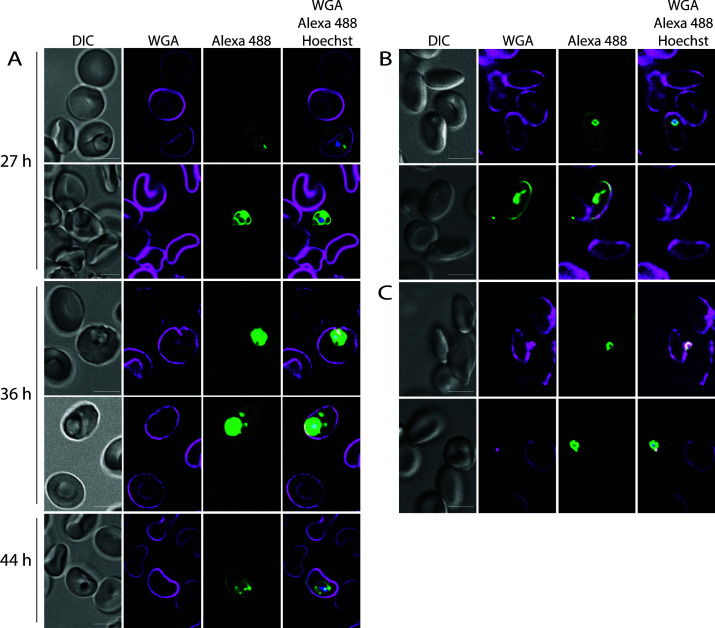
Timing of phospholipid transfer from the parasite to the erythrocyte plasma membrane. (**A**) Metabolic labeling of *Plasmodium falciparum* parasites was initiated immediately after invasion using a highly synchronized parasite culture. Samples were removed at the indicated time and fixed. (**B**) Phospholipid transfer to the host erythrocyte after invasion. Schizonts that had been labeled with propargylcholine for 72 h and prevented from egressing by the addition of ML10 were washed, thereby removing the ML10, and then allowed to egress and form new rings in the absence of propargylcholine. (**C**) Inhibition of invasion with cytochalasin D prevents transfer of phospholipids from the parasite to the host cell. Parasites were prepared as in (B) except that cytochalasin D was added to the culture of egressing parasites. Note the absence of staining of the erythrocyte plasma membrane. Scale bar: 5 µm.

### Transport of phospholipids to the erythrocyte plasma membrane can be detected from the trophozoite stage

As the preceding experiments were performed over one and a half cycles, it could not be determined at which time after invasion phosphatidylcholine transport of parasite phospholipids to the erythrocyte initiates. Therefore, we added propargylcholine to a culture of synchronized parasites immediately after the parasites had invaded the erythrocytes, thereby preventing any potential carryover of labeled phospholipid from the previous cycle. Labeling in the erythrocyte membrane was first detected during the trophozoite stage, approximately 36 h after invasion, indicating that phospholipid transfer to the host erythrocyte has initiated at this stage (Fig. 3A). Phospholipid transfer possibly initiates earlier, but the level of phospholipid may remain below the level of detection during that time; labeling of the erythrocyte plasma membrane was not robustly detected 27 h after invasion (Fig. 3A). The level of observed fluorescence detected gradually increased as the parasite matured, with labeling in the plasma membrane of erythrocytes containing schizonts brightest. These results are consistent with the findings that the alteration in the phospholipid content of the host erythrocyte plasma membrane changes most noticeably during the trophozoite stage (27).

### Phospholipids are transferred from the parasite to the host erythrocyte during or shortly after invasion

The origin of the membrane of the nascent PVM has been a subject of debate (6) although recent results support a model in which the erythrocyte plasma membrane constitutes the main source of phospholipids (7). However, the observed labeling of the host erythrocyte membrane could, in part, be the result of exchange of phospholipids during or immediately following the invasion process. As the labeling with propargylcholine allows the transfer of parasite phosphatidylcholine to the host erythrocyte to be investigated, we determined the distribution of parasite phosphatidylcholine around the time of invasion. We labeled synchronized parasites with propargylcholine for 72 h and then blocked egress with the cGMP-dependent kinase (PKG) inhibitor ML10 so that the timing of invasion of the labeled parasites could be carefully controlled (35, 38, 46). These parasites were isolated on a discontinuous Percoll gradient and then allowed to egress in the absence of ML10 and propargylcholine and invade fresh erythrocytes for 1 h. This revealed that the plasma membrane of the infected erythrocyte could be fluorescently labeled almost immediately after invasion (Fig. 3B; Fig. S4), indicating that transfer of phospholipids from the parasite to the host erythrocyte occurs during or very soon after invasion.

To narrow down the stage of the invasion process at which the transfer of phospholipids occurs, we blocked invasion by adding CD at the time ML10 was removed. In the presence of CD, the parasite binds to and reorients on the erythrocyte surface, forming a tight junction, but does not invade the host erythrocyte (47, 48); CD, therefore, inhibits one of the last steps prior to the initiation of invasion. In parasite cultures treated with CD, the parasites, but not the host erythrocytes, were brightly labeled with Alexa 488-azide, indicating that no phospholipid transfer had taken place (Fig. 3C). Hence, the transfer of phospholipids from the parasite to the erythrocyte plasma membrane occurs after the parasite has initiated invasion of the host erythrocyte.

### The tubovesicular network, but not Maurer’s clefts, contains parasite-derived phosphatidylcholine

**Fig 4:**
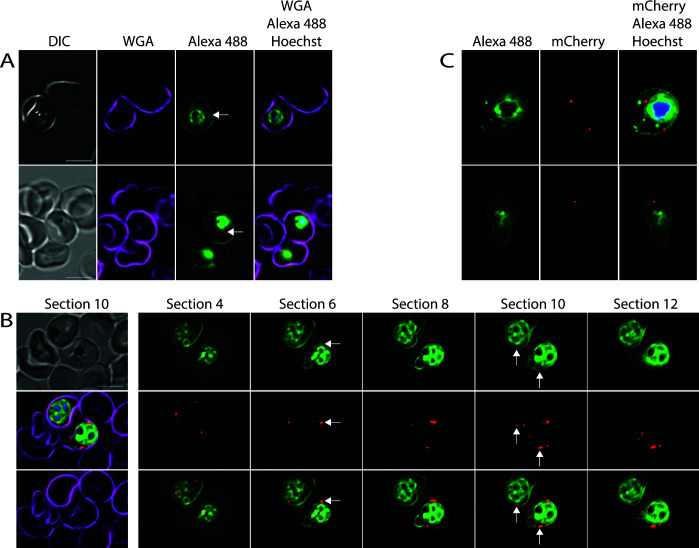
Labeling of the exomembrane system. (**A**) Labeled parasites displaying a stained tubovesicular network (arrow). (**B**) Maurer’s clefts are not labeled with Alexa 488-azide. *Plasmodium falciparum* parasites that produce a fusion of the Maurer’s cleft marker SBP1 with mCherry were metabolically labeled with propargylcholine for 72 h, fixed, and stained with Alexa 488-azide. The left-hand column shows section 10 of the deconvoluted z-series, and the other columns show the Alexa 488-azide labeling (top), mCherry labeling (middle), and the merge of the Alexa 488-azide and mCherry staining (bottom) of the indicated deconvolved sections. Arrows indicate select Maurer’s clefts that do not overlap with Alexa 488-azide staining. See Fig. S5 for further analysis of two of the Maurer’s clefts. (**C**) Confocal imaging of labeled parasites expressing SBP1-mCherry. Note the absence of colocalization of Alexa 488-azide and mCherry labeling in (B) and (C). For a 3D view of the parasites shown in (C), see Movies S1 and S2. Scale bar: 5 µm.

The parasite induces the formation of multiple internal membranes (Fig. 1A). Examination of the infected erythrocytes revealed that in some cases, loops protruding from the parasites could be detected. These loops likely represent the TVN (Fig. 4A) (49 – 52). This labeling indicates that this structure is also produced, at least in part, with phospholipids produced by the parasite. As the TVN is an extension of the PVM (50, 51, 53), it is likely that the PVM is also composed of parasite-derived phospholipids.

In the course of our investigation, we did not detect labeling that may represent Maurer’s clefts. To determine whether Maurer’s clefts contain parasite-derived lipids, we aimed to label parasites that produce the Maurer’s cleft protein SBP1 fused to mCherry. As mCherry remains fluorescent after fixation, Maurer’s clefts can be visualized in fixed cells without the need to permeabilize the erythrocyte for antibody staining. To produce such a strain, we transfected 3D7 parasites with a plasmid (pS156) that encodes an SBP1-mCherry fusion and repeated the staining in the resulting transfectants. Even when these parasites were labeled for one and a half cycles to maximize labeling, we were not able to detect Alexa 488-azide in Maurer’s clefts after staining (Fig. 4B), as was further confirmed by analysis of the intensity profile of two of the Maurer’s clefts in Fig. 4B (Fig. S5). Furthermore, we applied confocal microscopy and 3D reconstruction to look at this potential lack of colocalization in more detail. In this case, too, we were unable to detect staining in the Maurer’s clefts, even though the parasite and the erythrocyte surface were clearly labeled (Fig. 4C; Movies S1 and S2). As previous findings had indicated that there is likely to be a difference in the phospholipid content of the PVM and the Maurer’s clefts based on their different permeability after treatment with Streptolysin O (54), this finding is perhaps not surprising.

The absence of labeling from the Maurer’s cleft membrane, an internal membrane in the infected erythrocyte, further emphasizes the specificity of the labeling in the other internal and external membranes and underscores that the observed fluorescence reflects the presence of fluorescent phospholipid rather than nonspecific retention of propargylcholine or Alexa 488-azide in membranes or nonspecific transfer of propargylphosphatidylcholine between membranes during the preparation of the samples.

## DISCUSSION

Together, these experiments reveal that phosphatidylcholine produced by malaria parasites is transported from the parasite throughout the infected erythrocyte. Parasite-derived phosphatidylcholine was detected in the TVN (and, hence, is very likely to be present in the PVM, as the two membranes are physically connected) and the erythrocyte membrane. Transfer of phospholipids to the erythrocyte was detected immediately after invasion, although no transfer could be detected in the ring stage, phospholipid transfer from the parasite to the erythrocyte could be detected at the trophozoite stage. Interestingly, the Maurer’s clefts did not contain any detectable fluorescence after labeling, indicating that these structures are not produced using parasite phosphatidylcholine. The origin of the Maurer’s clefts has remained unclear; early suggestions that they are derived from the PVM (or even joined to the PVM) were contradicted by the finding that the PVM and Maurer’s clefts differ in their sensitivity to streptolysin O (54), indicating that their membrane compositions differ. Our results indicate that these compartments may form very soon after invasion of the erythrocyte and not be produced with parasite phospholipids.

The mechanism of the transport of phospholipids through the host erythrocyte remains to be discovered. As phosphatidylcholine is synthesized in the ER of the parasite (20), it is transported through the secretory pathway of the parasite to the parasite plasma membrane. From there, it must be transported through the aqueous lumen of the parasitophorous vacuole to the PVM—this could potentially occur through membrane contact sites between the parasite plasma membrane and the PVM (55)—from where the phospholipid has to be transported to the erythrocyte plasma membrane. The mechanism by which this occurs is not obvious. One candidate for a phospholipid transporter is the J dot, which carries cholesterol (56) but to date has not been shown to contain phospholipids. Alternatively, phospholipids could be carried by a phospholipid transfer protein (57). However, in contrast to the role of Maurer’s clefts in transport of proteins to the RBC plasma membrane, it is clear that Maurer’s clefts do not form an intermediate stage for transport of phosphatidylcholine to the surface of the erythrocyte, as no parasite-derived phosphatidylcholine was detected in Maurer’s clefts. The inhibition of transfer of the phosphatidylcholine from the parasite to the host cell in the presence of Brefeldin A (which appeared to trap the phospholipid in an internal organelle, mostly likely the ER) and the actin polymerization inhibitor cytochalasin D indicate that the phospholipid is produced in the ER and that actin polymerization is required for the distribution of the lipid throughout the infected erythrocyte.

Previous studies have shown that the phospholipid composition in the host erythrocyte plasma membrane changes the biophysical properties and fatty acid composition of the phospholipids of the infected erythrocyte plasma membrane (27, 30, 31). Using the biochemical methods employed, it could not be distinguished whether this was the result of transport of parasite-derived lipids to the membrane or whether this reflected modification of host lipids. Our finding that parasite-derived phosphatidylcholine is transported to the host erythrocyte plasma membrane likely indicates that the change in membrane lipids is the result of the addition of parasite phospholipids to the erythrocyte plasma membrane. Hence, the modification of the properties of the host erythrocyte is likely to be altered by the parasite not only solely by the export of proteins but also through the export of phospholipids.

## SUPPLEMENTAL MATERIAL

Figure S1 msphere.00131-23-s0001.pdf Titration of propargylcholine.

Supplemental figure legends msphere.00131-23-s0002.docx Legends for supplemental figures and movies.

Figure S2 msphere.00131-23-s0003.pdf Short-term labelling of erythrocytes infected with *Plasmodium falciparum*.

Figure S3 msphere.00131-23-s0004.pdf Effect of Brefeldin A and cytochalasin D on the transport of parasite-derived phospholipids to the host erythrocyte.

Figure S4 msphere.00131-23-s0005.pdf Analysis of membrane staining in erythrocytes containing recently invaded parasites.

Figure S5 msphere.00131-23-s0006.pdf Intensity profiles of the Alexa 488 fluorescence (representing parasite-derived phosphatidyl-choline) and the SBP1-mCherry fluorescence (representing Maurer's clefts) in infected erythrocyte shown in Fig. 4B.

Movie S1 msphere.00131-23-s0007.avi 3D reconstruction of the infected erythrocyte shown in the top panel of Fig. 4C.

Movie S2 msphere.00131-23-s0008.avi 3D reconstruction of the infected erythrocyte shown in the bottom panel of Fig. 4C.

ASM does not own the copyrights to Supplemental Material that may be linked to, or accessed through, an article. The authors have granted ASM a non-exclusive, world-wide license to publish the Supplemental Material files. Please contact the corresponding author directly for reuse.
