# Supplementary figures and images for: Distribution of malaria parasite-derived phosphatidylcholine in the infected erythrocyte
Source: mSphere. 2023 Aug 22;8(5):e00131-23. doi: 10.1128/msphere.00131-23 (PMC10597409; doi:10.1128/msphere.00131-23)

Supplementary Figure 1

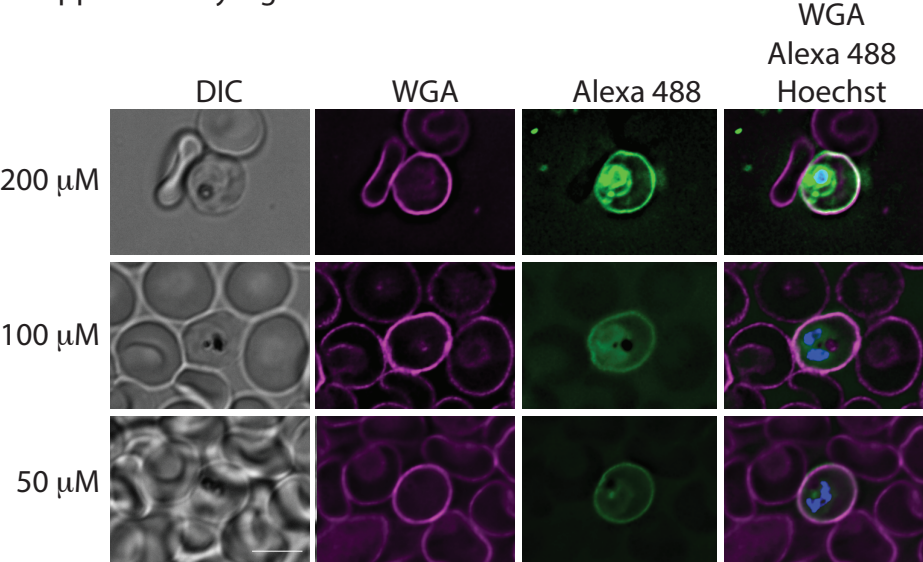

Supplement: Figure S1 — Titration of propargylcholine. [file msphere.00131-23-s0001.pdf]

Supplementary Figure 2

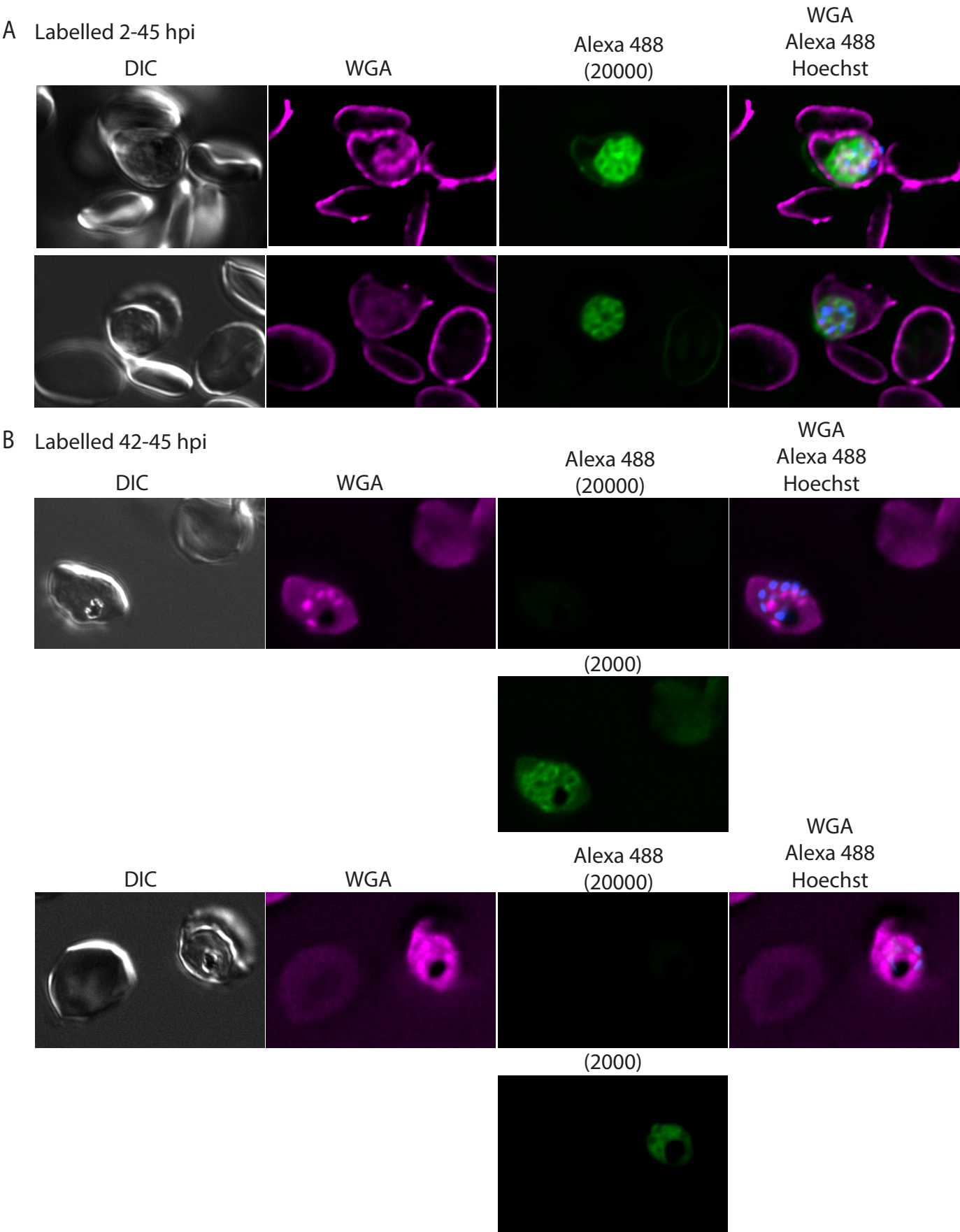

Supplement: Figure S2 — Short-term labelling of erythrocytes infected with Plasmodium falciparum. [file msphere.00131-23-s0003.pdf]

Supplementary Figure 3

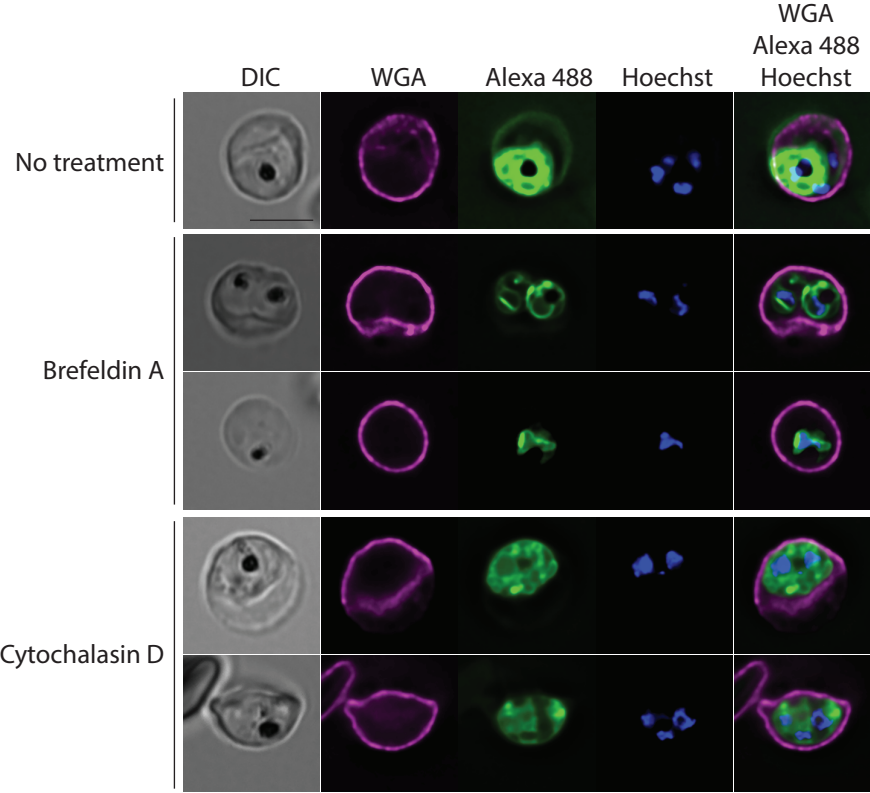

Supplement: Figure S3 — Effect of Brefeldin A and cytochalasin D on the transport of parasite-derived phospholipids to the host erythrocyte. [file msphere.00131-23-s0004.pdf]

Supplementary information 4

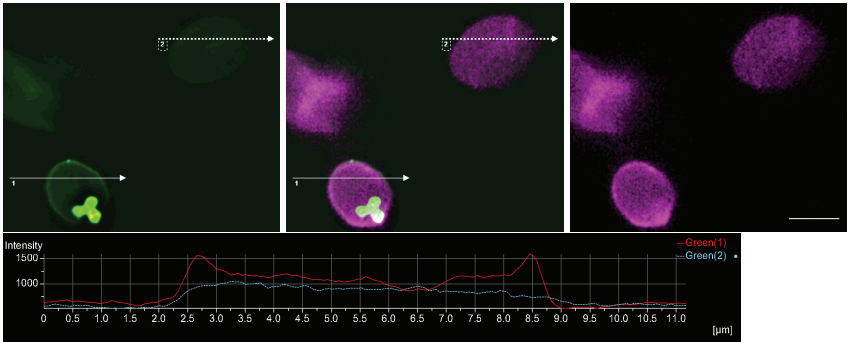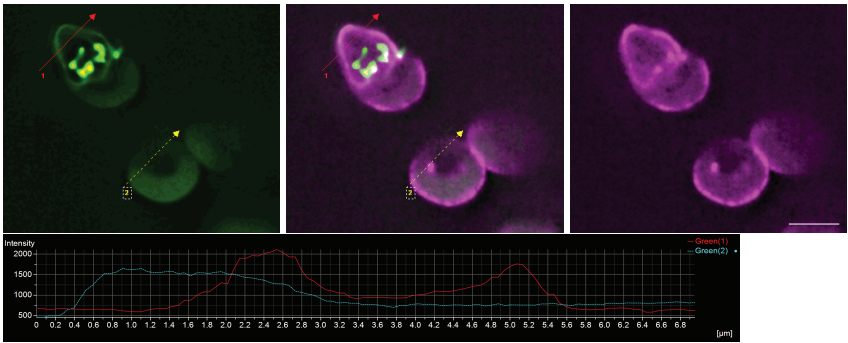

Supplement: Figure S4 — Analysis of membrane staining in erythrocytes containing recently invaded parasites. [file msphere.00131-23-s0005.pdf]

Supplementary Figure 5

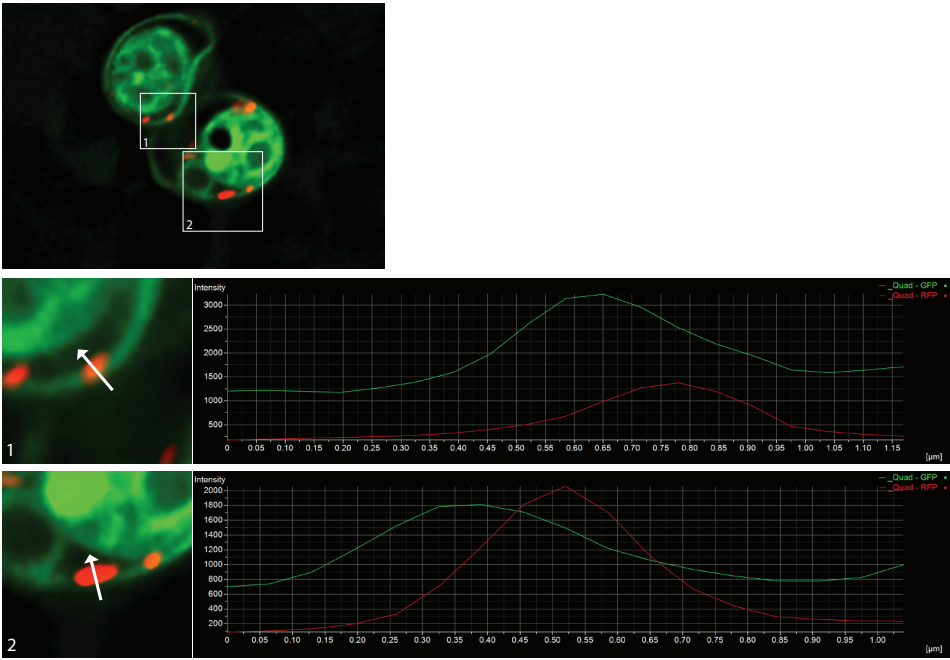

Supplement: Figure S5 — Intensity profiles of the Alexa 488 fluorescence (representing parasite-derived phosphatidyl-choline) and the SBP1-mCherry fluorescence (representing Maurer's clefts) in infected erythrocyte shown in Fig. 4B. [file msphere.00131-23-s0006.pdf]
