# Supplementary material for: Distribution of malaria parasite-derived phosphatidylcholine in the infected erythrocyte
Source: mSphere. 2023 Aug 22;8(5):e00131-23. doi: 10.1128/msphere.00131-23 (PMC10597409; doi:10.1128/msphere.00131-23)
Supplement: Supplemental figure legends — Legends for supplemental figures and movies. [file msphere.00131-23-s0002.docx]

Supplementary Figure 1: Titration of propargylcholine. Cultures of infected erythrocytes were incubated in the presence of the indicated concentration of propargylcholine for 72 hours. The erythrocytes were then fixed and labelled as described. Scale bar: 5 µm.

Supplementary Figure 2: Short-term labelling of erythrocytes infected with *Plasmodium falciparum*. Cultures of *P. falciparum*-infected erythrocytes were incubated in the presence of propargylcholine starting either 2 hours after invasion (A) or 42 hours after invasion (B). The culture was fixed and labelled 45 hours after invasion. The parasites were imaged under identical conditions and the obtained images were analyzed using FIJI. The Alexa 488-azide staining is shown using a contrast range of 150-20000 and in the case of the parasites labelled from 42 hours post-invasion, also using a contrast range of 150-2000 (the latter higher contrast image allows visualisation of weakly fluorescent cellular structures). Note the significantly lower brightness of the parasites labelled for only 3 hours.

Supplementary Figure 3: Effect of Brefeldin A and cytochalasin D on the transport of parasite-derived phospholipids to the host erythrocyte. Synchronized parasites were incubated in the presence of propargylcholine and 0.1% ethanol (solvent control; top), 1.25 µM Brefeldin A (middle) or 2 µM cytochalasin D (bottom) starting at the early trophozoite stage. The culture was fixed and labelled when the parasites without inhibitor had reached the schizont stage. Note the absence of staining of the host erythrocyte in the presence of either inhibitor. Scale bar: 5 µm.

Supplementary Figure 4: Analysis of membrane staining in erythrocytes containing recently invaded parasites. Brightness of erythrocyte membrane in deconvolved images of infected and uninfected erythrocytes was analyzed using the line intensity tool of the Nikon Elements-NIS software. Shown in each panel are fluorescence intensity plots of an infected erythrocyte (line 1) and an uninfected erythrocyte (line 2). Shown below is the intensity of the green signal along the line shown in the panel. Scale bar: 5 µm.

Supplementary Figure 5: Intensity profiles of the Alexa 488 fluorescence (representing parasite-derived phosphatidyl-choline) and the SBP1-mCherry fluorescence (representing Maurer’s clefts) in infected erythrocyte shown in Fig 4B. Boxes in top panels correspond to zoomed-in regions shown in below. Direction of Arrows indicate direction of traces of the intensity profiles are shown on the right-hand side. Note the lack of increased intensity of green (Alexa 488-labeled phospholipid) fluorescence at the points of maximum red (mCherry-SBP1) fluorescence, indicating that the Maurer’s cleft marker does not appear to colocalize with parasite-derived phosphatidyl-choline.

Supplementary movie 1: 3D reconstruction of the infected erythrocyte shown in the top panel of Figure 4C.

Supplementary movie 2: 3D reconstruction of the infected erythrocyte shown in the bottom panel of Figure 4C.
